# Stresses affect inbreeding depression in complex ways: disentangling stress-specific genetic effects from effects of initial size in plants

**DOI:** 10.1038/s41437-021-00454-5

**Published:** 2021-06-29

**Authors:** Tobias M. Sandner, Diethart Matthies, Donald M. Waller

**Affiliations:** 1 Plant Ecology, Department of Biology, Philipps-Universität Marburg, Marburg, Germany; 2 Department of Botany, University of Wisconsin – Madison, Madison, WI USA

**Keywords:** Genetic variation, Evolutionary ecology, Plant ecology

## Abstract

The magnitude of inbreeding depression (ID) varies unpredictably among environments. ID often increases in stressful environments suggesting that these expose more deleterious alleles to selection or increase their effects. More simply, ID could increase under conditions that amplify phenotypic variation (CV²), e.g., by accentuating size hierarchies among plants. These mechanisms are difficult to distinguish when stress increases both ID and phenotypic variation. We grew in- and outbred progeny of *Mimulus guttatus* under six abiotic stress treatments (control, waterlogging, drought, nutrient deficiency, copper addition, and clipping) with and without competition by the grass *Poa palustris*. ID differed greatly among stress treatments with *δ* varying from 7% (control) to 61% (waterlogging) but did not consistently increase with stress intensity. *Poa* competition increased ID under nutrient deficiency but not other stresses. Analyzing effects of initial size on performance of outbred plants suggests that under some conditions (low N, clipping) competition increased ID by amplifying initial size differences. In other cases (e.g., high ID under waterlogging), particular environments amplified the deleterious genetic effects of inbreeding suggesting differential gene expression. Interestingly, conditions that increased the phenotypic variability of inbred progeny regularly increased ID whereas variability among outbred progeny showed no relationship to ID. Our study reconciles the stress- and phenotypic variability hypotheses by demonstrating how specific conditions (rather than stress per se) act to increase ID. Analyzing CV² separately in inbred and outbred progeny while including effects of initial plant size improve our ability to predict how ID and gene expression vary across environments.

## Introduction

When related individuals mate, the fitness of the resulting inbred offspring usually declines relative to outcrossed offspring. This inbreeding depression (‘ID’) is predominantly caused by increases in homozygosity which increase the expression of deleterious recessive alleles (Charlesworth and Willis 2009). Estimating the magnitude of inbreeding depression is of central importance in conservation and evolutionary biology. Inbreeding effects tend to occur across all life history stages affecting both individual fitness and the overall viability of small, inbred populations (Hedrick and Kalinowsky 2000; Keller and Waller 2002; O’Grady et al. 2006). Inbreeding also operates to both facilitate and constrain plant and animal breeding (Weigel 2001; González et al. 2014). The almost universal presence of ID under inbreeding acts to favor self-incompatibility and other mechanisms that enhance or enforce outcrossing, in particular when ID exceeds in magnitude the transmission advantage of selfing (Darwin 1878; Barrett 2010; Carleial et al. 2017).

Although near universal, the magnitudes of ID often vary depending on the trait measured, the life history stage examined (Husband and Schemske 1996; Angeloni et al. 2011), and the environment it is measured in (Cheptou and Donohue 2011). This variation makes it impossible to ascribe any single value of ID to a particular species or population. It would thus be useful to generate theory allowing us to predict how conditions at some particular life history stage or in some environment likely affect ID (Yun and Agrawal 2014). In particular, are levels of environment-dependent inbreeding depression (or ‘EDID’ – Cheptou and Donohue 2011) predictable, or do they simply reflect idiosyncratic responses to particular conditions?

Two hypotheses have emerged to account for how inbreeding effects tend to vary in response to environmental conditions. It has long been observed that more stressful conditions can increase how much ID is observed (e.g., Wright 1922; Dudash 1990; Armbruster and Reed 2005; Reed et al. 2012). This *stress hypothesis* posits that more inbred individuals are intrinsically more susceptible to stress, increasing ID in more stressful environments. That is, that genetic deficiencies exposed by inbreeding become more deleterious as conditions become more stressful (Armbruster and Reed 2005; Cheptou and Donohue 2011; Reed et al. 2012). This could reflect either that effects of particular deleterious, mostly recessive mutations (the major cause of ID) increase under stressful conditions or that more such deleterious mutations emerge to be expressed under stress (Kondrashov and Houle 1997). Such responses clearly involve gene × environment (GxE) interactions. For example, inbreeding can reduce levels of plant defense, increasing herbivory or how well plants can adapt to stress via phenotypic plasticity (e.g., Campbell et al. 2014; Sandner and Matthies 2018; Schrieber et al. 2019). Although the stress hypothesis has intuitive appeal and is widely popular, empirical studies do not provide universal support. In fact, several studies show that ID can show little relation to stress or even increase in more benign environments (e.g., Waller et al. 2008; Cheptou and Donohue 2011; Sandner and Matthies 2016).

As an alternative and more parsimonious explanation for why ID tends to increase in certain environments, Waller et al. (2008) introduced the *phenotypic variability hypothesis*. They pointed out that any environmental conditions that amplify the amount of phenotypic variation present in a population should also increase the opportunity for inbreeding depression to be expressed. To implement this idea, they borrowed Crow’s (1958) derivation showing that the ‘opportunity for selection’ can be operationally measured as the phenotypic coefficient of variation squared (CV²), i.e., (standard deviation/mean)². This quantity sets an upper limit for how much selection can occur in any given environment in one generation (see Waples 2020 for refinements). Under this phenotypic variability hypothesis, ID increases whenever environmental conditions increase the opportunity for selection, which may be either the stressful or the more benign environment. One way environments affect the expression of ID is by affecting the density-dependence of survival (Yun and Agrawal 2014). Environments also affect ID by affecting size hierarchies that develop from differential growth within populations of plants (Schmitt and Ehrhardt 1990; Waller et al. 2008; Sandner and Matthies 2016). Particularly in plants, these differences in growth and reproduction are important as plant size and seed production often vary by orders of magnitude. Results from some studies provide support for the phenotypic variability hypothesis (Waller et al. 2008; Reed et al. 2012; Sandner and Matthies 2016). Other studies, however, find weak to no support (Sandner and Matthies 2017; Rehling et al. 2019). In any event, the phenotypic variability hypothesis is more explicit and more parsimonious than the stress hypothesis, because it does not require any particular GxE interactions to operate, allowing it to serve as a reasonable null-hypothesis against which we can test alternative hypotheses invoking more complex GxE mechanisms.

Given that we find mixed support for both the stress and phenotypic variability hypotheses, should we abandon the endeavor to seek generalities or a theory of EDID? Alternatively, can we combine these two hypotheses in some way to provide a more general predictive model of how environments affect ID? We clearly face difficulties in discriminating between the stress and phenotypic variability hypotheses if some stressful environments boost both phenotypic variation and levels of ID being expressed via additional genetic mechanisms. We must therefore be careful to assess how environments affect both phenotypic variation and ID under a variety of conditions.

### How do environments affect phenotypic variation?

**Fig. 1:**
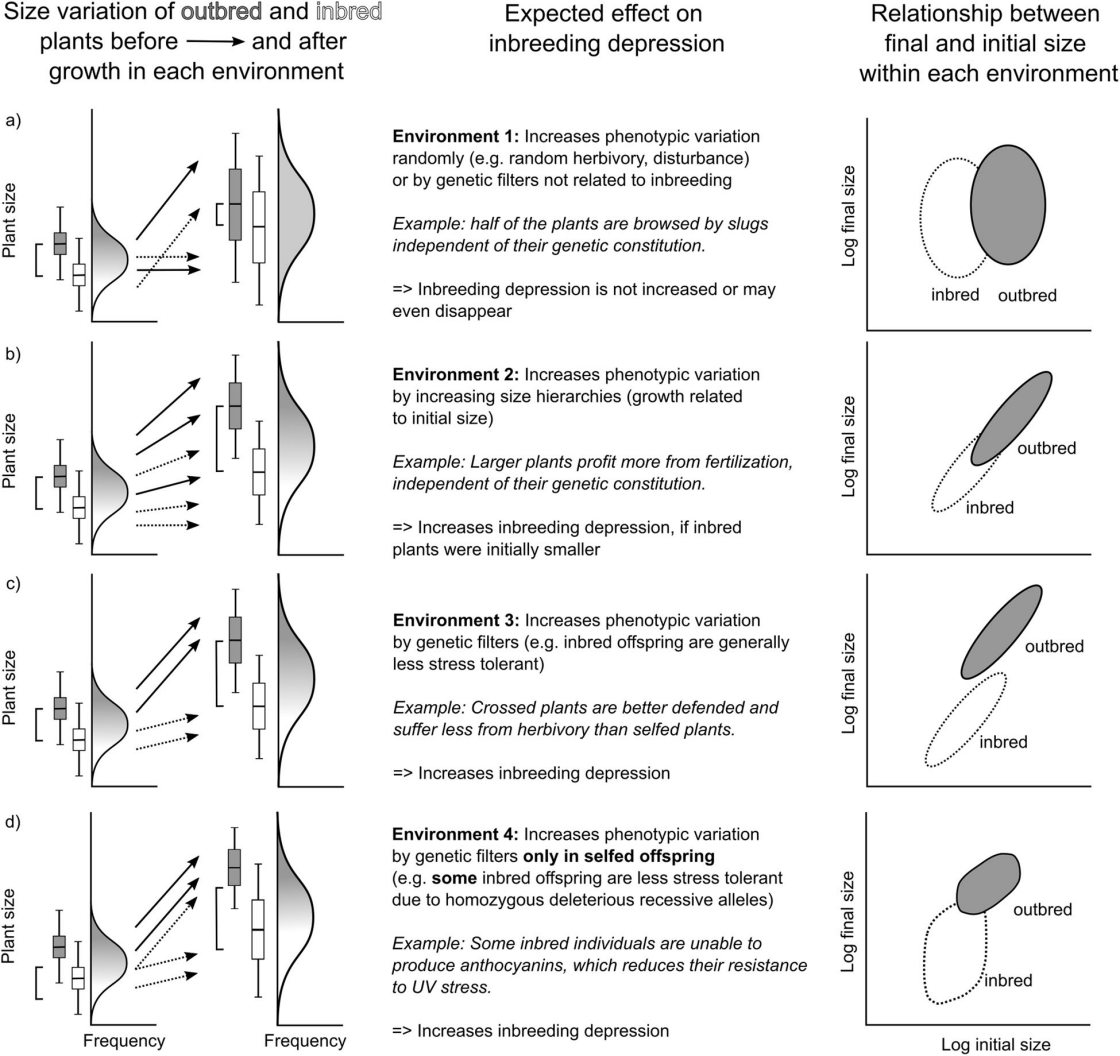
Four ways in which an environment could increase levels of phenotypic variation among individuals. Histograms show hypothetical size distributions of the population before (left) and after (right) growth in a given environment. Arrows illustrate the change in size of outbred (continuous lines) and inbred (broken lines) individuals due to the environment. Boxplots illustrate the size distributions of outbred (gray) and inbred (white) individuals in the population; brackets ([) indicate the difference in their means, i.e., inbreeding depression. The graphs on the right side of the figure illustrate for each environment possible relationships between initial and final plant size (note log scales of the axes)—different growth functions may, however, lead to similar patterns—see “Introduction”.

Environmental conditions can increase phenotypic variation among plants in at least four ways with each affecting ID in a different way (Fig. 1).

1. If random environmental effects increase phenotypic variation in ways unrelated to genotype or plant size (e.g., via disturbance or herbivory), CV² can increase without affecting ID (Fig. 1a).
2. Some environments might increase CV² by affecting growth (e.g., nutrient-rich sites). If selfed and outcrossed progeny follow similar growth curves, ID could increase along with plant size as growth amplifies initial differences in size (Fig. 1b). This would be the case if both selfed and outcrossed progeny fall on the same line regressing (log) final size on (log) initial size (Fig. 1b). In such cases, how much an environment increases ID simply reflects how strongly initial size determines final plant size in this environment, i.e., the slope of log size on log initial size. If relative growth rates (RGR) increase (or decline) with plant size, we would see more complex size-dependent environmental effects (not shown). If outbred seedlings were initially larger, ID and CV² would thus both be enhanced or both be reduced, depending on the relationship between RGR and plant size. For example, slightly larger plants often competitively suppress the growth of smaller neighbors by overtopping them or having more roots. Conversely, limits on growth (e.g., constraints on growth within pots or herbivores preferring to eat larger plants) could reduce CV² and ID over time.
3. Environments might increase both levels of phenotypic variability and ID, reflecting specific GxE effects. For example, selfed and outcrossed progeny could grow along parallel growth trajectories that differ in intercept (reflecting consistently higher fitness in the outcrossed group—Fig. 1c). Instead of a simple difference in starting capital (case 2, Fig. 1b), here some of the ID results from some intrinsic genetic difference affecting performance at all sizes/stages in that specific environment (e.g., outbred plants being better defended against herbivores or more competitive at all sizes).
4. Finally, it is also possible that some environmental conditions differentially affect some inbred progeny but not others. For example, some inbred individuals (homozygous at particular sites) may be less stress-tolerant or more susceptible to pathogens or herbivores, inflating phenotypic variation (CV²) only among inbred progeny in ways that could also increase ID (Fig. 1d).

This detailed framework allows us to pose additional questions as we seek to distinguish the two hypotheses of EDID outlined above (Table 1). Given that different environments generate different levels of ID (EDID exists, Q1), we can then test whether ID increases in more stressful environments (Q2, the *stress hypothesis*; Fox and Reed 2011). Alternatively, we can test whether ID increases in environments that increase phenotypic variation (Q3, the *phenotypic variability hypothesis*; Waller et al. 2008). However, it is necessary to distinguish between CV² in outbred and inbred progeny. Increased ID combined with higher variability among inbred but not outbred progeny (Q3a) would signal the presence of genetic mechanisms acting specifically in inbred individuals to increase their variability (Fig. 1d). We expect this to occur under conditions that preferentially increase variability among selfed progeny (e.g., by creating markedly inferior individuals). In contrast, results where environmental conditions increase ID and phenotypic variability in outbred progeny (Q3b) may support the simple phenotypic variability hypothesis (Fig. 1b).

**Table 1:** Questions related to size vs. genetic effects on inbreeding depression (ID).

Question	Test	Interpretation
Q1: Does ID differ between environments?	Is the environment × cross type interaction in a GLM of fitness significant?	If the magnitude of ID is affected by environments, these differences may reflect stress (Q2) and/or phenotypic variation (Q3).
Q2 – *Stress hypothesis:* Does ID increase as stress intensity increases over environments?	Does ID increase consistently with increases in stress intensity?	Increases of ID with stress intensity suggest that the number or expression of deleterious mutations increases under stress.
Q3 – *Phenotypic variability hypothesis*: Do environments that increase phenotypic variation increase ID?	Are regressions of ID vs. the mean CV² of selfed and outbred offspring significant?	Difficult to interpret given potential confounding with genetic effects—see Q’s 3a and 3b.
Q3a: Do environments that increase phenotypic variation among selfed progeny increase ID?	Are only regressions of ID vs. the CV² in inbred offspring significant?	Inbreeding generates specific genetic deficiencies that inflate the variation of inbred progeny in particular environments (see Fig. 1d).
Q3b: Do environments that increase phenotypic variation among outcrossed progeny increase ID?	Is a regression of ID vs. the CV² in outbred offspring significant across environments?	Yes: Environments consistently increase ID by increasing CV^2^. This may be due to effects on size variation (Fig. 1b → Q5) or due to genetic effects increasing both CV² and ID (Fig. 1c, d → Q7). No: See Q4a.
Q4a: Does ID increase in step with phenotypic variation in some environments?	Which environments increase both ID and CV² relative to the control environment?	In these particular cases, conditions may amplify ID by increasing phenotypic variation. This may be due to effects on size variation (Fig. 1b → Q5) or due to genetic effects increasing both CV² and ID (Fig. 1c, d → Q7).
Q4b: Are there environments that increase phenotypic variation but *not* ID?	Which environments cause ID to decline or remain the same relative to the control despite CV² increasing?	The existence of environments that increase phenotypic variation but not ID undermines the phenotypic variability hypothesis (cf. Fig. 1a).
Q5: Do some environments magnify initial size differences more strongly than others?	Do effects of initial size and environment interact in a GLM of fitness?	Treatments with higher slopes of log size on log initial size have the potential to increase ID. Compare with ID (Q6, Q7).
Q6: Are the effects of initial plant size sufficient to account for how much a given environment increases ID?	Does ID increase in step with increases in the sensitivity of final on initial plant size?	Environments falling on a 1:1 line of ID vs. slopes of log size on log initial size indicate amplification of initial size differences (cf. Fig. 1b).
Q7: Can we identify particular stressful conditions that magnify the genetic effects of ID?	(a) Examine outliers from the expected relationship between ID and the sensitivity of final on initial plant size. (b) Increased ID, but not CV², relative to the control.	(a) Genetic effects possibly increase ID in environments that magnify ID more than expected (given slopes of size on initial size—see Fig. 1c, d). (b) Genetic effects must act if ID increases under conditions where CV² does not (not shown in Fig. 1).

We propose particular ways to test each question, and how to interpret possible results. For more on specific questions like those related to how competition affects CV², variation in size, and ID, see text.

Parallel increases in CV² and ID in all (Q3b) or some (Q4a) environments can result from both size- and genetic effects (Fig. 1b–d). To test for these, it is important to control for the effects of initial seedling size (Questions 5–7 in Table 1). In a general linear model (GLM), the slopes relating final size of outbred offspring to initial size (within treatments) reflect the strength of initial size effects (Q5). This approach predicts how environments may amplify ID by amplifying initial differences in size (Fig. 1b). It echoes Sandner and Matthies’s (2016) use of a “coefficient of size depression” (see Supplement). If these slopes are high, we can then ask whether effects of initial size suffice to explain differences in ID (Q6), or whether some particular conditions enhance ID more than expected (Q7, Table 1). Such cases imply the presence of some genetic mechanism(s) that specifically reduce the ability of all (Fig. 1c) or some (Fig. 1d) inbred progeny to tolerate stress.

### Experimental approach

To assess how inbreeding depression responds to competition and other stresses, we applied five different abiotic stress treatments to selfed and outcrossed seedlings of *Mimulus guttatus* grown in the presence and absence of a grass competitor. Rather few studies have addressed how effects of competition and other stresses interact to affect ID either in animals (Keller et al. 2002; Yun and Agrawal 2014) or in plants (Kéry et al. 2000; Waller et al. 2008). Because competition is almost universal within plant populations and most abiotic stresses in nature occur in combination with competition, we should seek to understand how competition modulates the effects of stress on ID.

The abiotic stress treatments we chose cover a wide range of different functional plant responses to increase the chance of detecting recessive deleterious mutations affecting different kinds of stress response. Competition commonly increases size hierarchies among plants (Waller 1985; Weiner 1985; Schmitt et al. 1987). As the effects of competition fall disproportionately on smaller plants, dominance and suppression can magnify ID (Waller 1985; Schmitt and Ehrhardt 1990; Cheptou et al. 2001). However, competition can also affect how plants respond to the stress, altering ID. For example, nutrient deficiency can reduce ID if larger outbred plants quickly exhaust resources, limiting their own growth relative to that of smaller inbred plants (Sandner and Matthies 2016). A competitor might change this pattern by reducing available nutrients to similar levels in all pots. We thus expected ID to decrease under low N without competition, but to remain high under low N in the presence of a competitor. Generally, predictable interactions between interspecific competition and abiotic stresses can be expected when the two species differ in stress tolerance—an aspect we tried to minimize to be as general as possible by choosing a grass with a similar ecological niche as *M. guttatus*.

We ask the following questions: (a) Does ID differ among environments? (b) Is ID generally higher under more stressful conditions? (c) Does competition with a grass increase ID, at least in combination with some stresses? (d) Do levels of phenotypic variation serve to predict levels of ID? In particular, does the CV^2^ observed within outcrossed progeny predict ID (supporting the simple phenotypic variability hypothesis) or does the CV^2^ of selfed progeny better predict levels of ID among stresses? The latter case would support the idea that in certain environments genetic effects are an important source of both increased phenotypic variation and enhanced ID.

## Methods

### Study species

*Mimulus guttatus* DC. (Phrymaceae) is an annual or perennial plant species native to North America that is naturalizing along streams in Central Europe (Oberdorfer and Schwabe 2001; Truscott et al. 2006). It has large yellow zygomorphic flowers pollinated by large bees (Ivey and Carr 2005). Many populations are predominantly outcrossing, but the frequency of selfing differs strongly between populations in the natural range (Willis 1993; Ivey and Carr 2005, Brown and Kelly 2019). *Poa palustris* L. (Poaceae) is a perennial grass with an ecological niche similar to that of *M. guttatus* as indicated by their Ellenberg indicator values, which indicate that they typically occur under high light (both 7), very high humidity (both 9), and high nutrient conditions (7 for *P. palustris*; 6 for *M. guttatus* on an ordinal scale from 1 to 9, Ellenberg et al. 2001). Ellenberg indicator values ranging from 1 (habitat characterized by very low values of an environmental factor) to 9 (very high values) have been assigned to most plant species in Central Europe and describe the realized ecological niche of the species in Central Europe (Diekmann 2003). Like *M. guttatus*, *P. palustris* grows in wet meadows and on river banks of Central and Northern Europe and North America (Oberdorfer and Schwabe 2001) and the two species can occur in the same habitat (e.g., Hilbig 1975).

### Experimental crosses

Seeds of perennial *M. guttatus* were obtained from a commercial supplier (Jelitto, Schwarmstedt, Germany). No information is available on this population’s history, but we suspect no strong bottleneck or prior inbreeding as this population sustains considerable genetic variation (e.g., maternal differences in several traits—unpublished data). We also found substantial levels of ID in growth and reproduction (mean: 35%, see “Results”), which are similar in magnitude to those reported from natural populations in North America (18–32%, Willis 1993).

Fourteen parent plants were initially grown in 4 L pots filled with a 1:1 mixture of sand and commercial potting soil (TKS1, Floragard, Oldenburg) in a greenhouse. When plants started to flower, we covered them with a fine nylon mesh to exclude pollinators. We then emasculated matched pairs of flowers at the same node on each of ten plants. One flower of each pair was pollinated with self-pollen from another flower on the same plant while the other was cross-pollinated using a flower from a different plant. We marked both flowers for later seed collection.

### Stress treatments

We germinated the seeds resulting from these pollinations on wet filter paper. Two weeks later, we transplanted individual seedlings into 0.5 L pots (10 cm height) filled with a 1:1 mixture of sand and potting soil. The experiment consisted of 12 selfed and 12 outcrossed offspring from each of the 10 mother plants, resulting in 240 seedlings (23 later died during the experiment). Plants were grown in a climate chamber (75% relative humidity, 18 h of daylight, 22/17 °C). After 2 weeks, we randomly selected half the pots from each combination of mother plant and pollination type and sowed 0.5 g of seeds of *Poa palustris* from a commercial seed supplier (Rieger-Hoffmann, Blaufelden, Germany) into these pots as competitors. One week later, we measured the length and width of the leaf rosette of the *Mimulus* seedlings. Their product (length × width) estimates initial leaf area (we omitted initial size measurements for ~30 seedlings that had to be replaced in week 2). We randomly assigned one seedling of each combination of mother plant, pollination type, and competition to each of six abiotic stress treatments, resulting in 10 plants per treatment combination. Stress treatments were initiated 5 weeks after planting, i.e., 3 weeks after sowing the grass—its germination was thus not influenced by the stress treatments and all pots had similar competitor densities when abiotic stress was started. (1) *Control treatment*—pots were watered regularly to maintain a level of 2–3 cm in the pots. All pots received 20 ml of a 6.25 gL^−1^ fertilizer solution once a week (N, P, K = 14, 7, 14%, Hakaphos Gartenprofi, Compo, Wien). Growing conditions in the five stress treatments were similar except as noted. (2) *Copper addition*—pots received three 10 ml doses of a CuSO_4_ solution (10 g L^−1^) during the first week of the stress treatments, corresponding to 200 mg Cu per kg soil. This is about 400 times as much as the total copper applied with the fertilizer. (3) *Simulated herbivory*—*Mimulus* plants were cut above the lowest pair of leaves. The competing grass was cut at the same height. (4) *Nutrient deficiency*—pots received only water with no fertilizer. (5) *Flooding*—pots were kept in large troughs constantly immersed in water maintained at a level 0–1 cm above the soil. (6) *Drought*—plants experienced two drought periods during which they received no water for at least 4 days. Pots only received water when the *Mimulus* plants in them started to wilt. We chose the density of competing grass plants and levels of the five treatments based on our experience in a pilot study. These levels had strong effects on plant fitness but were not high enough to cause mortality. We randomized positions of all plants in the climate chamber regularly. We harvested all plants after 12 weeks of growth (i.e., 7 weeks of stress), counted the number of flowers, and measured aboveground biomass after drying plants at 80 °C to constant weight.

### Data analysis

We analyzed effects of pollination type, stress treatment and competition on initial leaf area, biomass and flower number of *Mimulus* and effects of pollination type and stress treatment on *Poa* biomass using linear mixed models in SPSS 22 with Satterthwaite’s approximation. We included maternal identity and the mother × pollination type interaction as random effects. We log-transformed initial leaf area, biomass, and flower number to homogenize variances and generate normally distributed residuals. As two plants had no flowers, 1 was added to flower number before log-transformation. Adding a smaller constant (0.1) gave more weight to the two outlier plants and weakened the stress × pollination interaction, but otherwise did not affect the results. Log-transforming the response variables also ensures that significant interactions of stress or competition with pollination type correspond to differences in relative ID between treatments (Q1 in Table 1; Lynch and Walsh 1998; Cheptou and Donohue 2011).

Within each treatment, we calculated the coefficient of inbreeding depression as (*w*_o_ – *w*_i_)/max (*w*_o_, *w*_i_), where *w*_o_ and *w*_i_ is the geometric mean of the biomass per treatment of outbred and inbred plants, respectively (Li et al. 2019).To test the hypothesis that ID increases as environments become more adverse (Q2), we followed Fox and Reed (2011) in estimating stress intensity as 1 − (biomass of the crossed plants in each environment/biomass of crossed plants in the control). We related ID to stress intensity by linear regression. To test the phenotypic variability hypothesis (Q3), we calculated three measures of the opportunity for selection (relative variance or squared coefficient of variation, CV²) within each treatment. We calculated CV^2^ as (SD/mean)^2^ from untransformed biomasses separately for the crossed and selfed offspring within each treatment cell, and also averaged over both groups within treatments. We analyzed how ID covaried with the three measures of variability using linear regressions (Q3, Q3a, Q3b).

We studied the effect of the individual environments on both ID and CV² in comparison to the control environment, and in particular which environments may increase both ID and CV² (Q4a) and which may increase only CV² without increasing ID (Q4b). However, as we did not have replicates for each combination of seed family × pollination type per environment, we could not formally test these relationships. We used ANOVA to test if CV² in a treatment was influenced by competition. To test if the relationship between ID and the CV² of inbred offspring differed between treatments with and without competition, we used a general linear model with type I SS relating ID in a treatment to competition, CV², and their interaction.

To understand how initial seedling size affected subsequent performance, we focused on offspring from cross-pollination. Analyzing just the outcrossed progeny allowed us to estimate how environments would influence ID if crossed and selfed plants differed only in initial size (i.e., in the absence of inbred genetic effects—Sandner and Matthies 2016, Q5). We applied a general linear model (Type III SS) to relate final plant biomass to initial size, stress treatment, competition, and their interactions. We calculated the slopes of linear regressions of log final size on log initial size for each stress × competition combination. These slopes describe the expected ID resulting from size differences alone. They are highly correlated with Sandner and Matthies’s (2016) “coefficient of size depression” (*r* = 0.88). Using slopes here is mathematically more robust, however, and easier to understand (see Supplement). We related these slopes of linear regressions of log final size on log initial size in a treatment to ID using linear regression. High ID could be caused by environmental amplification of initial size differences (Q6), whereas deviations above (or below) the 1:1 line suggest that ID was higher (or lower) than expected from effects on size variation alone (Q7).

## Results

### Effects of inbreeding and competition

Inbreeding depression (ID) emerged quickly in the *Mimulus guttatus* seedlings. After 3 weeks of growth (i.e., before the abiotic stresses were applied and after 1 week of competition), the leaf area of inbred progeny was 47.0% smaller than that of outcrossed progeny (5.9 [SE: +1.1, −1.0] vs 11.2 [SE: +2.1, −1.8] cm²; *F*_1,8.8_ = 9.02, *p* = 0.015). Effects of competition were also evident at this point as seedlings grown with *Poa* were already 30.3% smaller than seedlings growing alone (*F*_1,173.7_ = 4.85, *p* = 0.029). These effects of inbreeding and competition persisted. At harvest, inbreeding reduced the number of flowers and biomass to similar degrees (*δ* = 32.3 and 37.8%, Table 2—reflecting their high correlation: *r* = 0.83, *p* < 0.001). In pots with the competing grass, the many *Poa palustris* plants collectively produced considerably more biomass than the single *Mimulus* plant (means per pot: 4.7 ± 0.3 vs. 1.2 ± 0.1 g). Competition from the grass greatly reduced *M. guttatus* biomass (mean: 67.6%) and flower number (mean: 59.8%) relative to treatments without competition.

**Table 2:** Results of linear mixed models of the effects of stress treatment, competition, and pollination on aboveground biomass and the number of flowers of *Mimulus guttatus*.

		Biomass		Flower number	
Source of variation	df	*F*	*p*	*F*	*p*
Stress treatment	5	**7.94**	**<0.001**	**20.467**	**<0.001**
Competition	1	**156.79**	**<0.001**	**84.032**	**<0.001**
Pollination type	1	**11.06**	**0.004**	**7.358**	**0.024**
Stress × competition	5	1.21	0.307	1.768	0.122
Stress × pollination	5	**2.34**	**0.044**	**2.544**	**0.030**
Competition × pollination	1	1.03	0.312	0.488	0.486
Stress × comp. × poll.	5	1.62	0.157	1.101	0.362

Significant effects (at *p* < 0.05) are bolded. Denominator degrees of freedom were close to 18 (biomass) or 9 (flower number) for the effect of pollination type and between 176 and 181 for the other effects. Mother plant and mother × pollination type were included as random effects in the models, with variances of 0 (M) and 0.010 ± 0.006 (M × P) for biomass and 0.002 ± 0.006 (M) and 0.010 ± 0.009 (M × P) for flower number.

### Effects of abiotic stresses

**Fig. 2:**
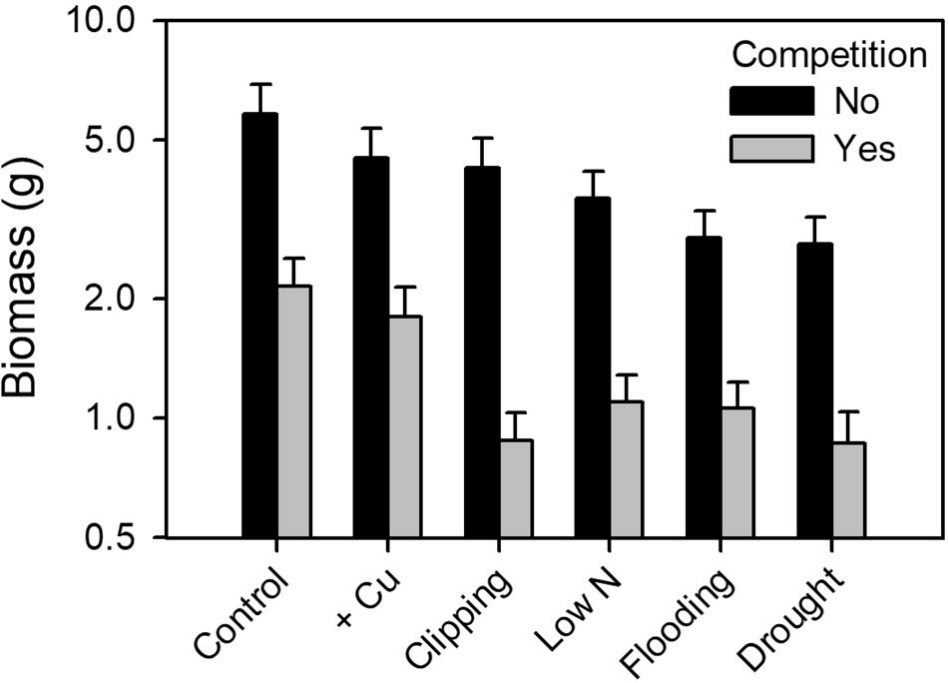
Effects of stress treatment on the biomass of *M. guttatus* with and without competition by a grass. Stress treatments are sorted in the order of increasing reduction of biomass under no competition compared to the control. Bars show means + 1 SE.

The stress treatments strongly depressed growth of *Mimulus* (Table 2). While copper addition weakly affected plant size (−18.9%) and reproduction (−14.7%), the drought treatment reduced plant biomass by 55.8% and flower number by 77.6% relative to the control. Drought was thus the strongest stress (Fig. 2). The type of stress also influenced *Poa* biomass (*F*_5,97_ = 11.5, *p* = 1.03 × 10^−8^), but this was only due to a strong reduction by nutrient deficiency (mean = 2.4 ± 0.3 g), while across all other treatments grass biomass was similarly high (mean = 5.4 ± 0.3 g).

**Fig. 3:**
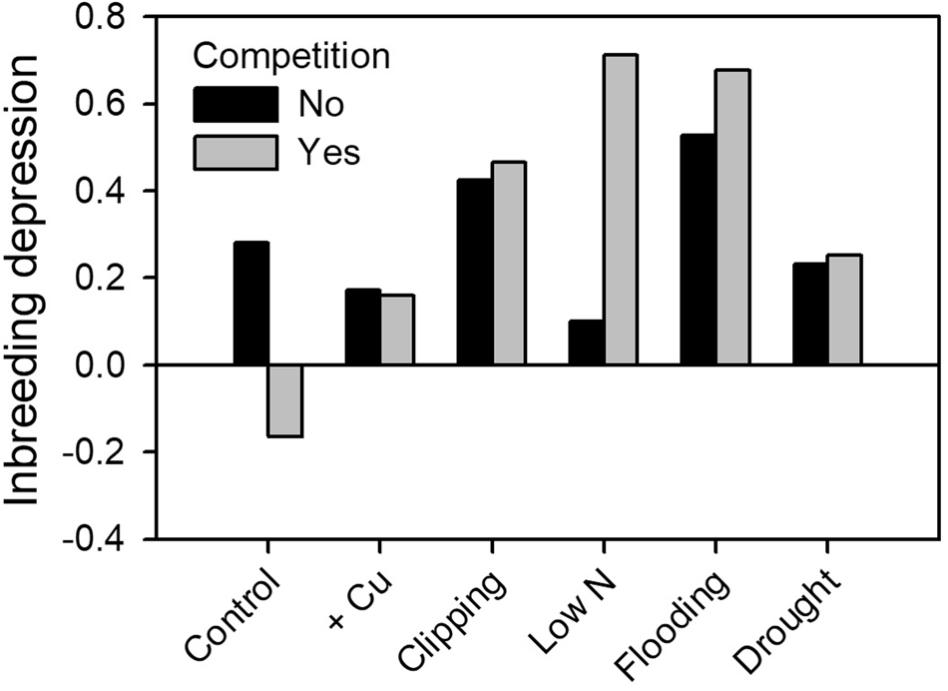
Effects of stress treatment and competition with the grass *Poa palustris* on the coefficient of inbreeding depression (*δ*) of the biomass of *M. guttatus* plants. Stress treatments are sorted in order of increasing reduction of plant biomass relative to the control (see Fig. 2).

Inbreeding affected final biomass differentially among the stress treatments (Table 2, Fig. 3, corresponding to Q1 in Table 1). Combining pots with and without competition, ID was highest under the flooding treatment (*δ* = 61.0%), in the simulated herbivory treatment (*δ* = 44.7%), and under low N (49.2%). ID was much lower in the control treatment (*δ* = 7.3%) and modest under weak copper stress (*δ* = 16.6%), and when drought severely limited *Mimulus* growth (*δ* = 24.1%). Across treatments, levels of ID were unrelated to stress intensity that was estimated from the decline in fitness of outcrossed progeny within treatments (*r* = 0.079, *p* = 0.81, Q2). Although competition did not consistently affect the magnitude of ID (Table 2), ID was high under low N in the presence of *Poa* competition but nearly absent without competition (Figs. 3 and S2). Similar patterns emerged for flower number (Fig. S3).

### Effects of phenotypic variation and size hierarchies

**Fig. 4:**
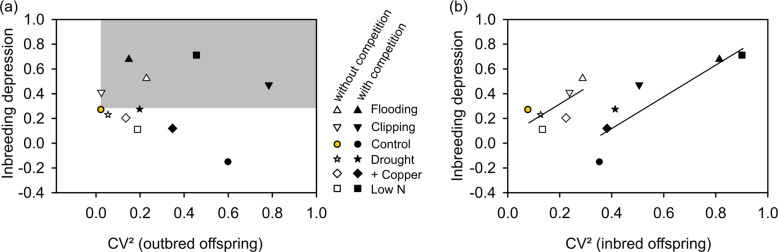
Relationship between levels of inbreeding depression and opportunities for selection (CV²) for outcrossed and inbred offspring. Panels show how ID was related to CV² measured within treatment cells (stress × competition) in either (**a**) outcrossed or (**b**) inbred progeny. Lines show linear regressions within groups with and without competition (*N* = 6 each). Note that competition with a grass (filled symbols) significantly increased CV² in outcrossed (*p* = 0.014) and selfed offspring (*p* = 0.004) but had inconsistent effects on ID. The shaded gray area in (**a**) shows four treatments that increased both CV² and ID relative to the control, supporting the phenotypic variability hypothesis, in contrast to the seven others that did not.

Levels of ID were not significantly related to phenotypic variation estimated as the mean CV² of selfed and outcrossed progeny within a given environment (*r* = 0.37, *p* = 0.23, Q3). This pattern, however, blurs the sharp distinction in predictability present within the separate outcrossed and inbred progeny groups. Levels of ID were unrelated to estimates of CV² within the outcrossed progeny growing in a particular environment (*r* = −0.042, *p* = 0.896, Q3b) with only four environments showing parallel increases in both ID and CV² in relation to the control (indicated by symbols in the gray box in Fig. 4a, Q4a), and five environments increasing CV² but not ID (symbols below the gray box in Fig. 4a, Q4b). In contrast, ID showed a strong consistent response to increases in CV² within the selfed progeny across environments (*r* = 0.65, *p* = 0.023, Q3a). Competition from *Poa* strongly increased CV² within crossed offspring across environments (*F*_1,10_ = 8.8, *p* = 0.014) and within selfed offspring (*F*_1,10_ = 14.0, *p* = 0.004). The relationship between ID and CV² in selfed offspring was stronger (*r* = 0.86, *F*_1,8_ = 23.9, *p* = 0.0012) when the effects of competition were taken into account and separate lines were fitted for plants grown with and without competition (Fig. 4b; no significant CV² × competition interaction, *F*_1,8_ = 0.003, *p* = 0.96). In sum, we found evidence for genetic effects in inbred plants on phenotypic variation rather than pure environmental effects on phenotypic variation.

**Fig. 5:**
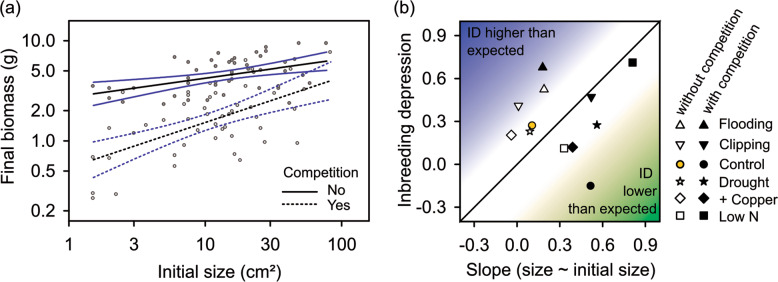
**Effects of initial plant size on subsequent plant growth and on levels of inbreeding depression in different environments. a** Effects of initial size on the biomass of outbred plants grown with (broken lines) and without (continuous lines) grass competitors. Lines are estimated using separate linear regressions (*N* = 47 and 48, respectively) and 95% confidence intervals are shown. **b** Relationship between levels of inbreeding depression and slopes of regressions of final size on initial size of outbred plants (our measure of expected ID caused only by effects of initial size, see Supplement, Fig. S1) across the six stress treatments and two levels of competition. Note that treatments with competition (filled symbols) have on average higher slopes than those without (open symbols), matching the effect shown in (**a**), but do not exhibit higher ID. Blue color indicates that ID was higher than predicted by the effects of initial size differences, whereas green indicates that ID was lower than expected.

To test how environments influenced size variation among plants in the absence of genetic effects of inbreeding, we analyzed results from just the outcrossed progeny. In a general linear model both initial size and competition exerted strong effects on the eventual size of outcrossed offspring (*F*_1,71_ = 17.4 and 23.1, respectively, both *p* < 0.001). Initial seedling size affected final plant size more strongly in the presence of *Poa* competition (*F*_1,71_ = 6.797, *p* = 0.011, Fig. 5a, Q5). Here, neither stress treatment (*F*_5,71_ = 0.058, *p* = 0.715) nor the other interactions (*p* > 0.6) were significant. Across all treatments, our estimates of ID were unrelated to slopes of individual regressions of final size on initial size within treatments (our measure of how much initial size effects affect ID – *r* = 0.096, *p* = 0.766, Fig. 5b). ID estimates fell along the expected line in some treatments (Q6), but were lower than expected in the control with competition treatment and higher than expected in the flooding and non-competition clipping treatments (Fig. 5b, Q7).

## Discussion

Both *Mimulus* growth and levels of ID varied greatly in response to the competition and abiotic stress treatments, reinforcing how widespread EDID is (Q1, Table 1). Under classical expectations, ID increases with stress (Fox and Reed 2011; Rosche et al. 2018). However, despite having suitably high levels of variation and testing multiple kinds of stress, we found no evidence that ID increases in response to greater intensities of stress (Q2). Other recent studies similarly failed to find that levels of stress predict levels of ID (Waller et al. 2008; Sandner and Matthies 2016; Carleial et al. 2017; Rehling et al. 2019). In the current study, ID peaked when plants were waterlogged or were subject to herbivory or nutrient deficiency in the presence of *Poa* competitors. There may be different reasons for the high ID in each of these conditions. In the following, we will first discuss potential effects of stresses on size differences among plants which may modify the amount of ID. We then discuss environmental effects on ID probably related to genetic differences between crossed and selfed offspring in how they respond to flooding.

### Effects of stress on phenotypic variability

As explored in the “Introduction”, various stresses affect levels of phenotypic variation and resulting size hierarchies in ways that can affect how ID is expressed. Under the phenotypic variability hypothesis, one would expect ID to increase with Crow’s (1958) opportunity for selection which sets an upper limit to how much selection can occur (Waller et al. 2008). We estimated the opportunity for selection as either the mean CV² for selfed and outcrossed progeny grown within each environment (following Waller et al. 2008) or the CV² observed only among outcrossed progeny. Neither variable predicted ID well in our experiment. Only 4 of 11 stressful environmental treatments increased both ID and the opportunity for selection in outcrossed offspring relative to the control (Fig. 4a). High ID could result from increased phenotypic variation in these four environments, but five other environments increased CV² but not ID (corresponding to the hypothetical environment in Fig. 1a). These results fail to support the simple phenotypic variability hypothesis (Q3b). In contrast, CV² within inbred progeny did accurately predict levels of ID (Q3a), particularly when competitive effects were accounted for (Fig. 4b). This result suggests that genetic factors that increase levels of ID amplify size differences particularly among the inbred plants (Fig. 1d). Such effects are consistent with the idea that inbreeding acts particularly to restrict growth in certain inbred individuals. This is congruent with the dominance mechanism of ID where inbreeding generates variable levels of homozygosity among individuals (identity disequilibrium) and thus inbreeding effects from segregating deleterious mutations (Bierne et al. 2000; Charlesworth and Willis 2009; Charlesworth 2018). Recent sequencing work in *Mimulus guttatus* supports this deleterious recessive mechanism (Brown and Kelly 2019). We hypothesize that inbred progeny express more homozygous loci (or greater effects from those loci) in certain environments (after Kondrashov and Houle 1997), increasing inbreeding depression and phenotypic variation in parallel. As a corollary, selection against these deleterious recessive alleles (purging) should operate most efficiently under particular environmental conditions that increase both ID and the opportunity for selection against low-fitness inbred individuals.

### Effects of initial size on inbreeding depression

Although we focused on ID in size and flower number, the fitness of individuals also depends on seed viability and survival. These traits might show different patterns of environmentally determined inbreeding depression (EDID). However, this may be particularly relevant for animals, which vary little in adult size but strongly in fecundity and survival, whereas plant biomass, flower number, and seed production all tend to be closely correlated in herbaceous plants.

In the “Introduction”, we explored how to distinguish EDID due to environmental effects on plant size from EDID caused by G × E interactions (e.g., differences in stress response). Environments that increase ID by amplifying phenotypic variation should increase both ID and CV² among outbred plants (Q4a). Four environments fit this prediction (low N with competition, clipping with competition, and flooding with and without competition). However, as parallel increases in ID and CV² could also reflect genetic effects (Fig. 1c, d) we suggested comparing ID to how environments affect the amplification of initial size differences (again in outbred plants, Q6–7). Any environments increasing inbreeding effects beyond those predicted from early size effects suggest that genetic factors act to further amplify ID.

Inbreeding effects were evident after just 3 weeks of seedling growth in *Mimulus guttatus*. Substantial ID existed for leaf area then (47%). ID declined to 35% when we measured biomass and flower production after seven more weeks of growth. These levels of ID resemble those found in other experiments with *Mimulus* and other plant species (Willis 1993; Byers and Waller 1999) but are substantially lower than inbreeding effects can be in *M. guttatus* when lethal and sterile mutations are included (Brown and Kelly 2019). Because inbred plants were already smaller when the environmental stresses were initiated, we expected treatments that amplified size differences to generate higher inbreeding depression in final size, even if inbreeding did not affect subsequent plant growth trajectories. We evaluated these size effects by examining the dependence of log final plant size on log initial size—again in outbred progeny. This dependence measures the degree to which ID could be caused only by differences in initial size. Where ID and slopes were both high, size effects alone suffice to explain why ID increased in some treatments (Q6, e.g., low N and clipping with competition which fit Fig. 1b). In contrast, levels of ID increased more than expected among waterlogged plants and when plants were clipped in the absence of competition (Q7). Initial size may be unimportant under these conditions if larger plants lose their advantage by suddenly losing most of their roots (due to anoxia) or leaves (due to clipping or herbivory). High levels of ID under these conditions suggest that particular genetic inbreeding effects are expressed under these stresses. To statistically compare ID in an environment with size dependency of growth, replicated inbred and outbred replicates of several seed families should ideally be used within each environment to study EDID. We tested predictions from this study in a follow-up study with *Mimulus* plants grown under flooding. These results support our interpretation that size effects do not account for the high ID observed under flooding (Supplement, Fig. S4).

### Effects of competition on phenotypic variation and ID

Adding grass competition to the pots increased effects of initial leaf area on the final size of *Mimulus* (Q5). This accords with the common observation that competition disproportionately affects the growth of small individuals (Waller 1985; Schmitt and Ehrhardt 1990). However, differences in initial size hardly influenced final plant size in most treatments without *Poa* competition. One reason may be that *Mimulus* plants grown individually in pots may become limited by nutrients and space, limiting effects of initial size. However, an alternative explanation is that genetic differences among plants (e.g., in resource use efficiency or photosynthesis) affect plant size more strongly than initial size differences.

Although competition by *Poa* consistently increased both CV² and the slopes of final on initial plant size, it did not consistently increase ID in our experiment (Fig. 3). Similarly, interspecific competition did not consistently increase ID in several other studies (Kéry et al. 2000; Willi et al. 2007, Walisch et al. 2012; but see Cheptou et al. 2000). In contrast, many studies found high ID under intraspecific competition (e.g., Darwin 1878; Schmitt and Ehrhardt 1990; Eckert and Barrett 1994). This could reflect how size hierarchies tend to increase over time as larger individuals block light and take up more water and nutrients, suppressing the growth of smaller individuals via “dominance and suppression” (Weiner 1985; Schmitt et al. 1987). In fact, ID does tend to increase when in- and outbred individuals directly compete (Schmitt and Ehrhardt 1990; Cheptou et al. 2001; but see Li et al. 2019). Inbred individuals invading an outbred population thus face not only dominance and suppression but also the likely presence of stronger (outbred) competitors. Invoking a similar mechanism, Yun and Agrawal (2014) proposed that environments that increase negative effects of density on fitness will also increase ID. Here, inbred and outbred plants grew in separate pots, preventing direct competition. However, competition with a different species can also increase size hierarchies. If an individual of the focal species is small (e.g., due to inbreeding), individuals of the other species benefit from reduced competition, allowing them to suppress focal plants more strongly. This may explain why all our treatments with competition expressed high phenotypic variation.

Competition failed to increase ID under the control, copper, and drought treatments, perhaps reflecting size effects. Under the most severe (drought) stress, growth of both outcrossed and selfed *M. guttatus* plants was suppressed by the stress-tolerant grass. In contrast, under weak stresses, even inbred *M. guttatus* plants may have been large enough to compete effectively against *Poa*. Competition could act to increase ID mainly when competitors are large enough to compete effectively against most selfed progeny but not against larger outcrossed progeny. If so, such effects should also be manifest within populations of the same species (as observed by Schmitt and Ehrhardt 1990; but see Willi et al. 2007; Li et al. 2019). ID was strongest under competition combined with nutrient deficiency (Fig. 3). This may reflect high competition between *Mimulus* and *Poa* occurring in that treatment. We chose *Poa palustris* as a competitor because it grows in similar habitats as *M. guttatus*. However, stress treatments generally depressed growth more in *M. guttatus* than in *P. palustris*. Only nutrient deficiency appreciably reduced the growth of both *Poa* and *Mimulus*. The ability of *P. palustris* to thrive under stressful conditions could reflect some overall stress hardiness. Alternatively, the several *Poa* seedlings in each pot may have allowed them to collectively resist stresses better than the single *Mimulus* individual.

### Specific effects of flooding stress on inbreeding depression

Flooding strongly increased ID relative to the control in a manner unrelated to initial leaf area. This suggests that inbreeding exposed deleterious recessive alleles specifically related to flooding responses that are masked in the heterozygote state. Growth in waterlogged soils requires specific responses to anoxic conditions including physiological changes and producing adventitious roots above the soil surface (Colmer and Voesenek 2009). Recessive deleterious alleles affecting stress responses in any of these traits could lead to higher ID under waterlogging. These high levels of ID under waterlogging are surprising given that *Mimulus* typically grows in wet environments and that flooding affected plant growth less than drought. Several authors suggest that ID occurs especially under novel forms of stress as deleterious recessive alleles expressed only under those conditions would not be purged as consistently as alleles affecting growth under common conditions (Bijlsma et al. 1999; Cheptou and Donohue 2011; Sandner and Matthies 2016). However, an experiment designed to test this hypothesis failed to find higher ID under novel vs. familiar stresses in *Anthyllis vulneraria* (Rehling et al. 2019). This could reflect the fact that purging is inefficient under many conditions (Keller and Waller 2002; Glémin 2003). In any case, the commercial *Mimulus* seeds used in our experiment derive from plants grown under non-flooded conditions to enhance growth. Thus, any purging associated with flooded conditions has not occurred for several generations.

## Conclusions

The diverse stresses applied in these experiments affected plant performance and levels of inbreeding depression in several different ways. Neither abiotic stress nor competition generally increased ID. Rather, particular stresses acted to increase ID. The specific nature of these responses and the fact that variability only among the selfed progeny consistently enhanced ID suggest that particular deleterious alleles are differentially expressed within specific environments. Our study thus reconciles the stress- and the phenotypic variability hypotheses: Certain types of stress clearly increase ID (e.g., flooding here), but this is not a general attribute of stress. Rather, different inbreeding effects emerge under different kinds of stress, suggesting variation in which mutations are expressed and perhaps the population’s historic experience with that stress. In addition, particular environments can increase ID by enhancing phenotypic variation (competition at low N levels here) without specific genetic inbreeding effects. Our analytical framework distinguished these mechanisms by simultaneously assessing both how environments affect opportunities for selection (separately within progeny groups) and how size hierarchies develop within plant populations. Applying this framework to other systems should allow us to test its generality and ability to reliably discriminate effects of size from those of genetic factors on how inbreeding depression is expressed.

### Data archiving

Data available from the Dryad Digital Repository: 10.5061/dryad.vx0k6djrv.

## Supplementary information

Supplement
