# Supplementary material for: Stresses affect inbreeding depression in complex ways: disentangling stress-specific genetic effects from effects of initial size in plants
Source: Heredity (Edinb). 2021 Jun 29;127(4):347–56. doi: 10.1038/s41437-021-00454-5 (PMC8478953; doi:10.1038/s41437-021-00454-5)
Supplement: Supplementary file 1 — Supplement [file 41437_2021_454_MOESM1_ESM.docx]

**Sandner, TM, Matthies, D, Waller, DM (2021): Stresses affect inbreeding depression in complex ways: Disentangling stress-specific genetic effects from effects of initial size in plants. Heredity.**

**Supplement**

**The coefficient of size depression – an alternative approach to test effects of variability in initial size on inbreeding depression**

In the paper we used the slope of final size on initial size calculated within each treatment as a measure of the strength of the effect of initial size. Alternatively, one could also use the approach used by Sandner & Matthies (2016) and calculate “coefficients of size depression”. In the following we compare both approaches.

For the calculation of size depression, only offspring from cross pollinations were analyzed. Outcrossed plants were classified within each treatment x competition cell by their leaf area after three weeks relative to the median initial size as either initially large (larger than the median size) or initially small (smaller than the median size). We then calculated a coefficient of size depression (by analogy to the coefficient of inbreeding depression – Sandner & Matthies 2016) for the plants in each treatment from the mean biomass at harvest of initially large and small plants: (biomass_large_ – biomass_small_)/ biomass_large_. The “coefficient of size depression” thus represents a ‘pseudo-ID’, as it shows how large ID would be if selfed and crossed plants differed only in initial size, and not in their genetic constitution. However, the threshold to divide initially small and large plants is arbitrary – instead of dividing plants by half one could also divide the plants in three quantiles, ignore the intermediate plants in the center and use only the large and the small plants, as was done by Sandner & Matthies (2016), an approach that requires a larger number of replicates within each stress treatment.

Both measures of the effect of initial size on final plant size, the slopes and coefficients of size depression, were strongly correlated (Fig. S1a) and fulfilled the same purpose, leading to very similar results (compare Fig. S1b with Fig. 5b in the main text).


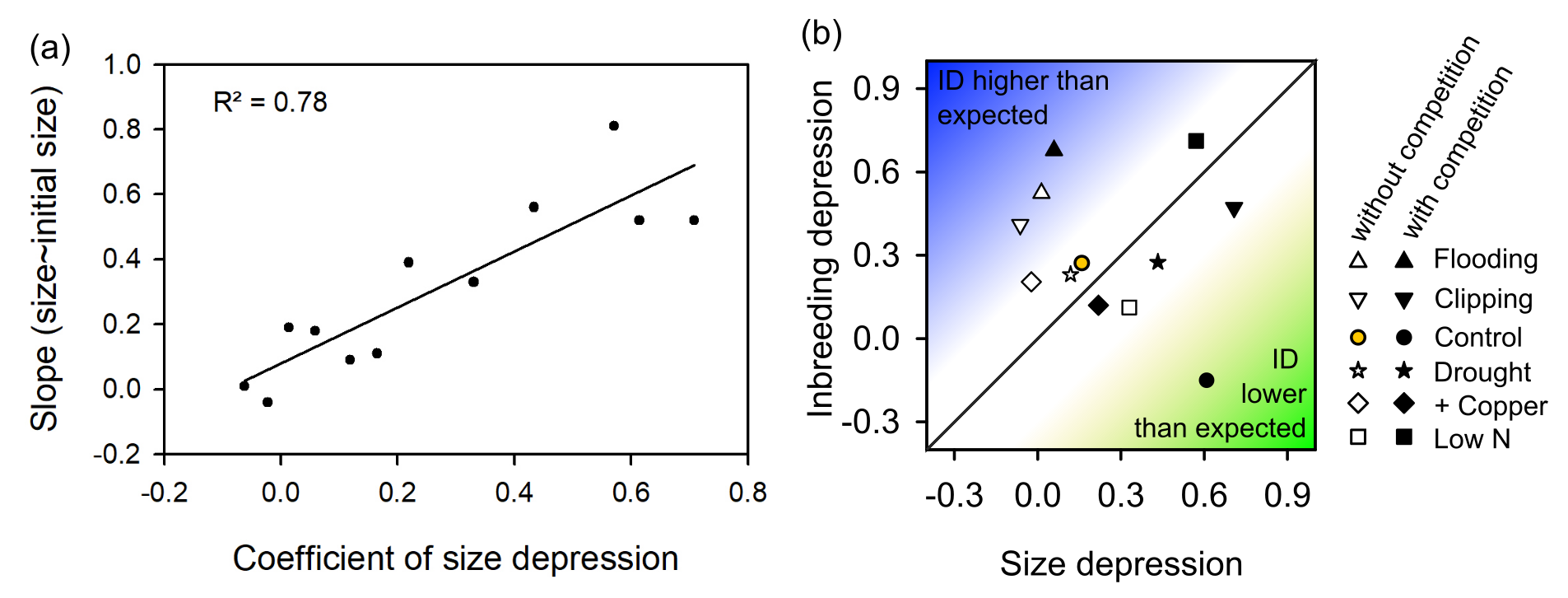


**Fig. S1:** Relationship between (a) slopes of regressions of log final size on log initial size calculated within each combination of stress x competition and the coefficient of size depression and (b) inbreeding depression and the coefficient of size depression. Note that the pattern and interpretation do not differ from those presented in the article using slopes of regressions of final on initial size (compare Fig. 5b).


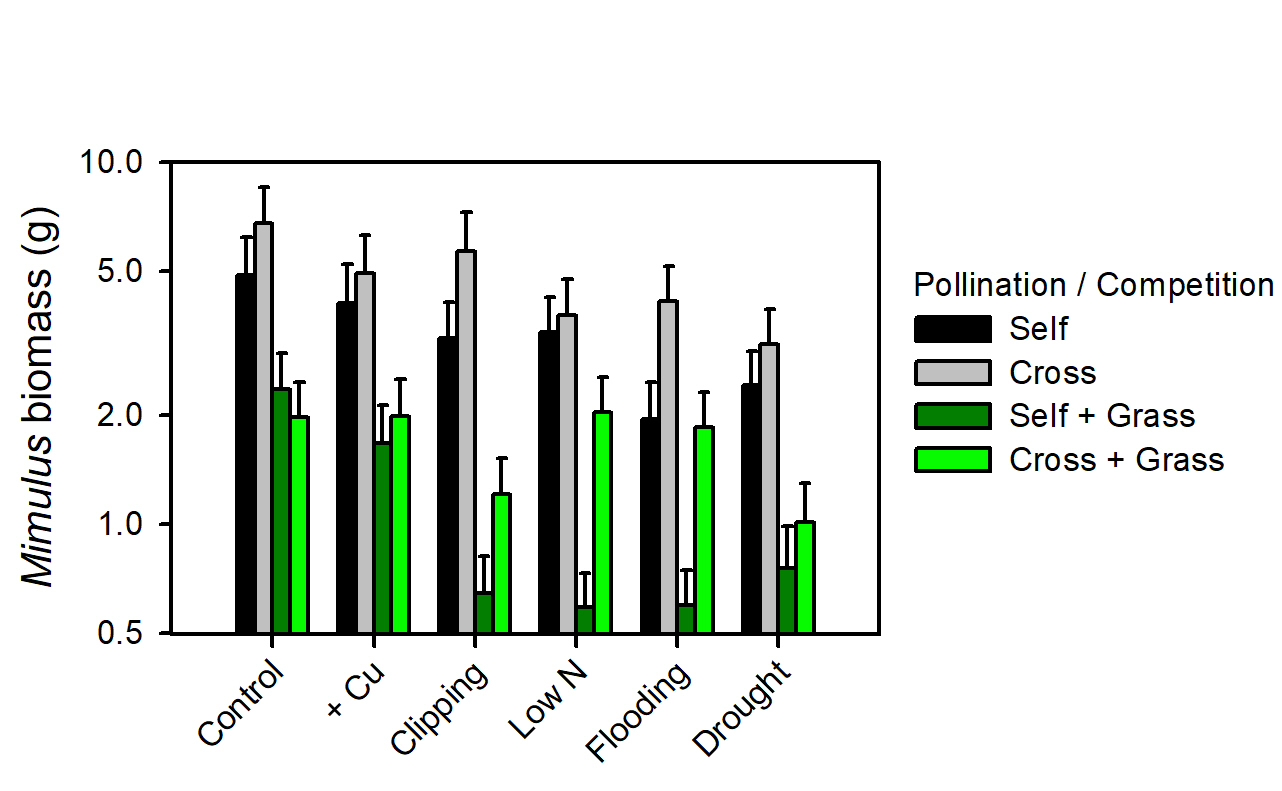


**Fig. S2:** Effects of stress treatment, pollination type and competition with the grass *Poa palustris* on the biomass of *Mimulus guttatus* plants. Stress treatments are sorted by increasing reduction of plant biomass compared to the control. Note that in the low N treatment inbreeding hardly affected plant size when no competitor was present, but the effect of inbreeding was pronounced in the presence of grass competition. Means + SE.


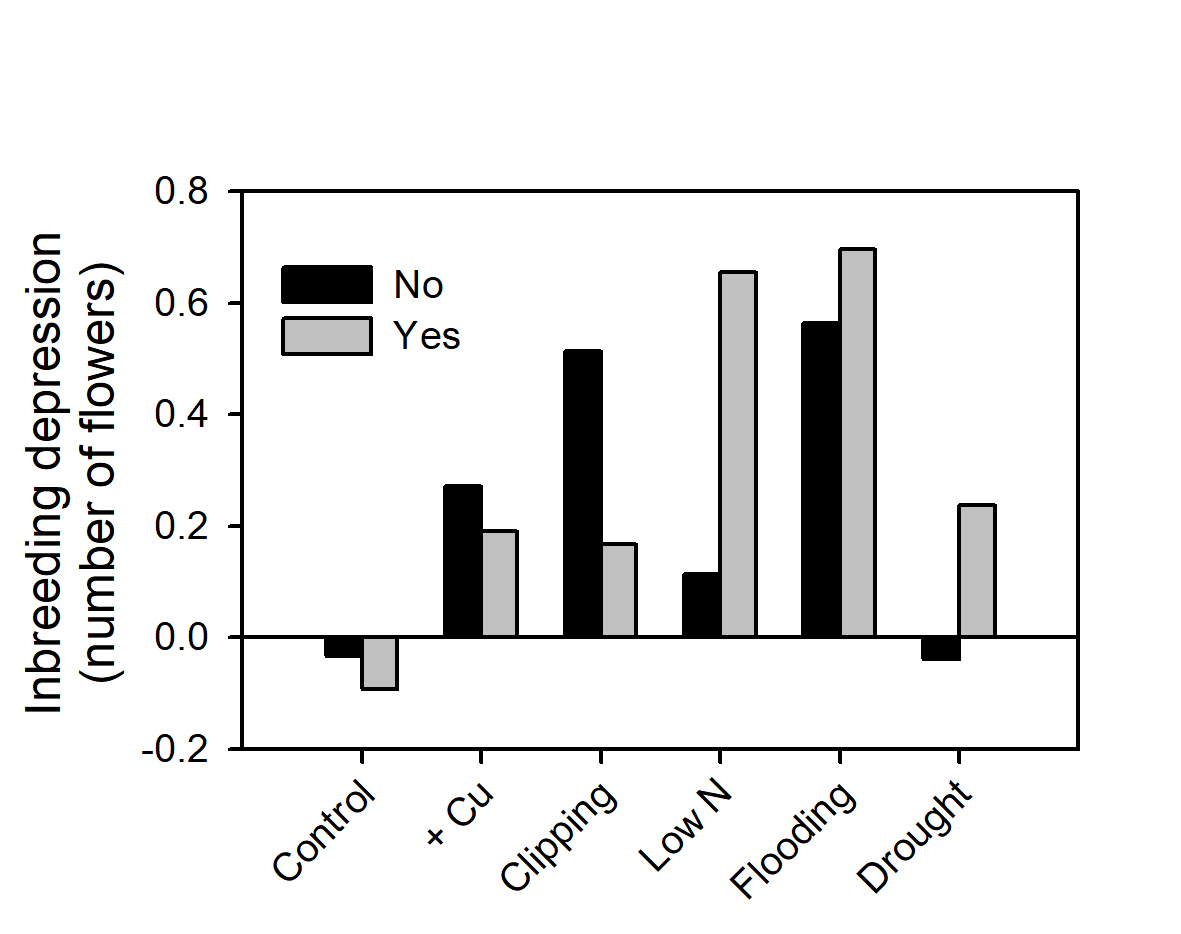


**Fig. S3:** Effects of stress treatment and competition with the grass *Poa palustris* on the coefficient of inbreeding depression (δ) of the number of flowers of *Mimulus guttatus* plants. Stress treatments are sorted by increasing reduction of plant biomass compared to the control, see Fig. 2.

Follow-up experiment to test size vs. genetic effects of flooding on ID

The results of the main experiment suggest that under flooding ID was increased not due to size-effects, but due to genetic effects. However, the design of the experiment did not allow to test the significance of the relationship between ID and CV within each environment. We thus performed a follow-up experiment exposing seedlings from the same pollination experiment to flooding and control conditions with higher replication.

Methods

In this follow-up experiment, we used seeds from the same pollinations as in the main experiment. Two seedlings from each combination of nine of the mother plants and pollination type (i.e. 36 genotypes) were vegetatively propagated to form 4 clonal replicates per seedling. Clonal propagates were cultivated as described for the plants in the main experiment. Five weeks after planting, daylength was increased from 12 to 16 h to induce the formation of flowering stems. Six weeks after clonal propagation, rosette diameter was measured as an estimate of initial plant size and flooding stress was started. Thus, two of the clonal replicates for each genotype were kept at water levels of 1-2 cm (control), and the other half were flooded with water up to the soil surface (i.e. 9.5 cm water level). Plants were harvested after 11 weeks of flooding and their flower number and above-ground biomass measured as fitness components.

Effects of flooding on ID were analysed with linear mixed models testing the effects of flooding, pollination and their interaction on log-transformed biomass and flower number, with mother plant, mother x pollination type and the genotype included as random factors. To differentiate between size-related and genetic effects of flooding on ID, we calculated the coefficient of inbreeding depression for each seed family under control and flooding conditions, and the opportunity for selection (CV²) separately for selfed and crossed offspring per maternal family within a treatment. We then tested with analyses of variance the effect of flooding on CV², and we tested with linear regressions for relationships between family level ID and CV² of selfed and crossed offspring. An increase of ID with the CV² of crossed offspring would suggest that ID may have increased only as a result of increasing phenotypic variation (Waller et al. 2008). In addition, we analysed with linear mixed models the effects of log initial size, stress and their interaction on log-transformed biomass and flower number of outbred offspring after 11 weeks, including mother plant as a random factor. A significant interaction between the effects of initial size and flooding on final size would indicate that growth curves differ between control and flooding environments, and a steeper slope of the relationship between final size and initial size under stress would indicate the potential of the environment to increase ID by size effects.

Results

Inbreeding depression was higher under flooding than under control conditions (biomass: F_1,33.9_ = 8.47, p = 0.006, flower number: F_1,34_ = 3.59, p = 0.067). The opportunity for selection (CV²) of biomass per seed family was significantly increased under flooding (0.078 ± 0.014 vs. 0.027 ± 0.014) for selfed offspring (F_1,16_ = 6.32, p = 0.023), but did not change in crossed offspring (F_1,16_ = 2.28, p = 0.151), and CV² of flower number did not change with flooding in selfed or crossed offspring (both F_1,16_ < 1, p > 0.3). ID per family was not related to the opportunity for selection (CV²) of crossed offspring (Fig. S4a; biomass: r = 0.10, p = 0.69, flower number: r = 0.02, p = 0.925) but tended to increase with CV² of selfed offspring (Fig. S4b; biomass: r = 0.36, p = 0.147), an effect that was significant only for flower number (r = 0.53, p = 0.025). Initial rosette diameter of outbred plants positively influenced final biomass (F_1,68.0_ = 5.83, p = 0.018) and flower number (F_1,63.9_ = 7.89, p = 0.007), but did not interact with the flooding treatment (F_1,65.6_ = 0.13, p = 0.72; F_1,61.8_ = 0.001, p = 0.98). Slopes of log size on log initial size were thus not significantly different between the two environments.


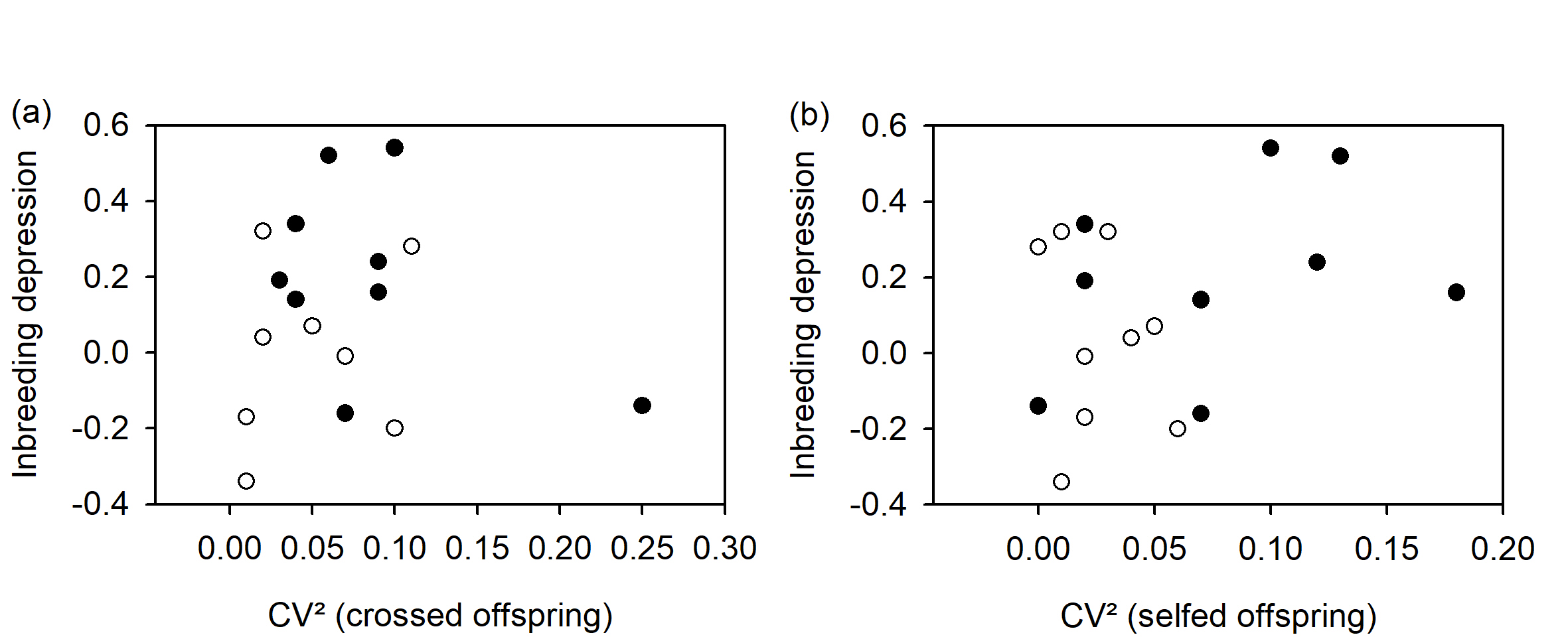


**Fig. S4:** Relationship between family-level inbreeding depression and opportunity for selection (CV²) in biomass measured under control (open circles) and flooded conditions (filled circles) in either (a) outcrossed or (b) self-pollinated progeny of each of 9 seed families. Both effects were not significant, see text.

Conclusions

The results suggest that, as already suggested by the main experiment, increased ID under flooding cannot be explained by different flooding effects on plants of different initial size. Slopes of the relationship between log final size and log initial size did not differ between control treatment and flooding, and the CV² of outbred offspring was not increased by flooding compared to control conditions. There is thus no reason to expect an increase in ID by flooding through size-mediated effects. Instead, the increase of ID under flooding can thus be attributed to other (GxE) effects. The same patterns were suggested by the results of the main experiment, but could only be tested for each specific environment with the higher replication for each combination of maternal family and pollination type within each environment in this follow-up experiment.
